# Supplementary material for: Structural insights into the mechanism of rhodopsin phosphodiesterase
Source: Nat Commun. 2020 Nov 5;11:5605. doi: 10.1038/s41467-020-19376-7 (PMC7644710; doi:10.1038/s41467-020-19376-7)
Supplement: Supplementary file 1 — Supplementary Information [file 41467_2020_19376_MOESM1_ESM.pdf]

Structural insights into the mechanism of rhodopsin phosphodiesterase

Supplementary Information

T. Ikuta *et al.*

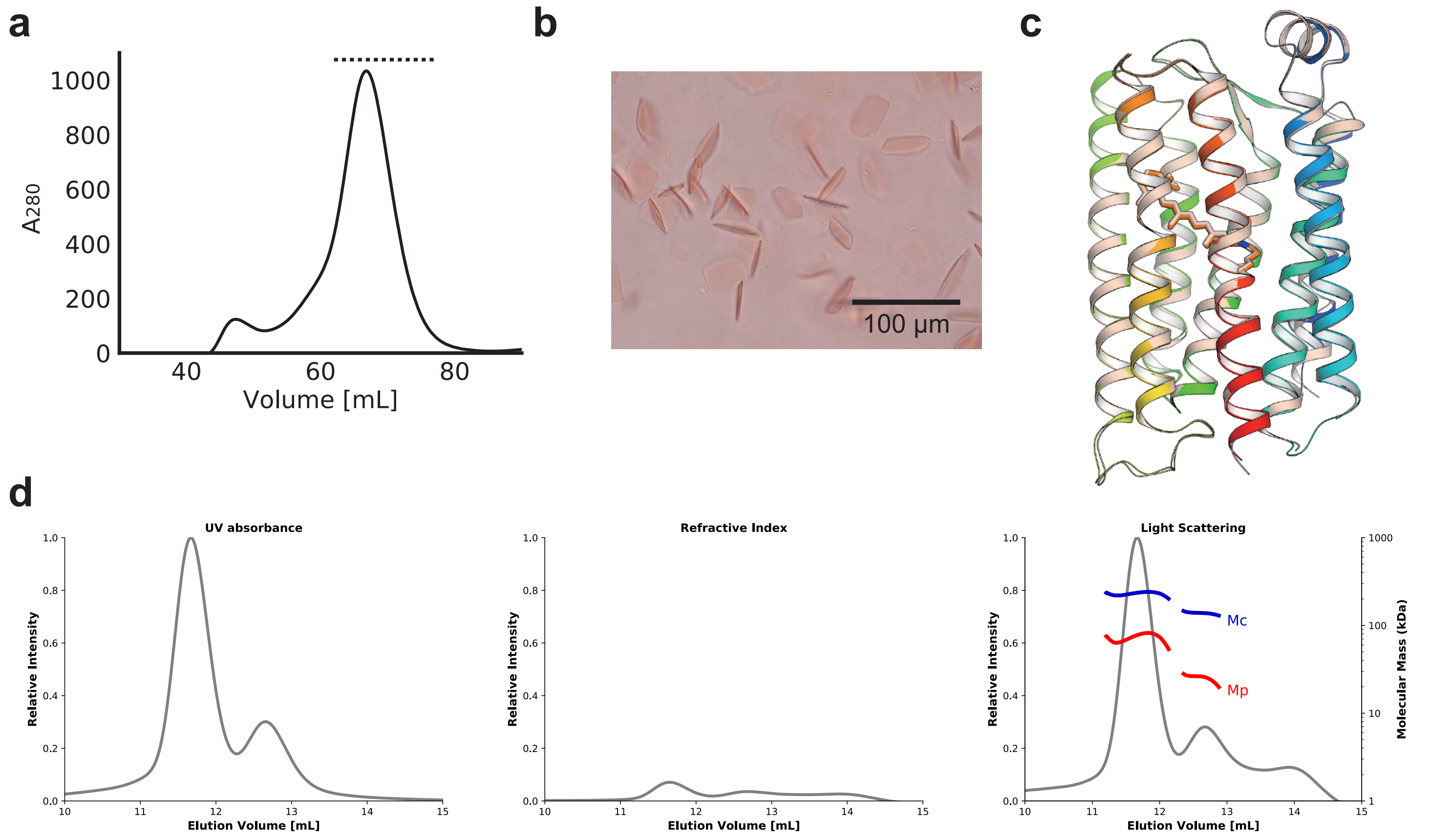

e

	Molecular Mass (kDa)	
	Former peak	Latter peak
Protein-detergent complex	231.3 (±0.107%)	139.9 (±0.131%)
Protein	72.99 (±0.104%)	26.38 (±0.133%)
Dimer (theoretical)	65.0	
Monomer (theoretical)	32.5	

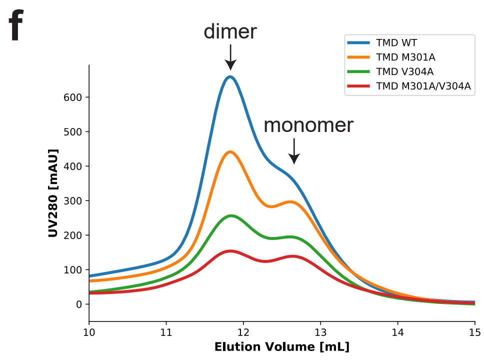

Supplementary Fig. 1 | Purification and crystallization of Rh-PDE-TMD. a, Gel filtration chromatogram of the purified Rh-PDE-TMD. Fractions with dashed lines were collected for the following crystallization. **b,** Crystals of Rh-PDE-TMD. **c,** Structural comparison between mol A and B. Mol A and B are colored rainbow and light orange, respectively. **d,** SEC–MALLS analysis of Rh-PDE TMD wild type. The three chromatograms show the readings from the UV absorption, refractive index, and light-scattering detectors. The traces were normalized to the peak maxima. The blue and red curves in the light-scattering chromatogram indicate the calculated molecular masses of the protein–detergent complex (Mc) and the protein (Mp), respectively. This experiment was performed once. **e,** Molecular mass values determined by the SEC–MALLS experiment with the standard errors on fitting or calculated from the amino acid sequence. The experimental protein mass was determined to be about 72.99 kDa at the former peak and 26.38 kDa at the latter peak, corresponding to the theoretical mass of Rh-PDE dimer and monomer, respectively. **f,** SEC chromatograms of purified Rh-PDE TMD mutants, using Superdex 200 increase 10/300 column. The monomer:dimer ratio is 1:2 in wild type while 1:1 in M301A/V304A mutant.

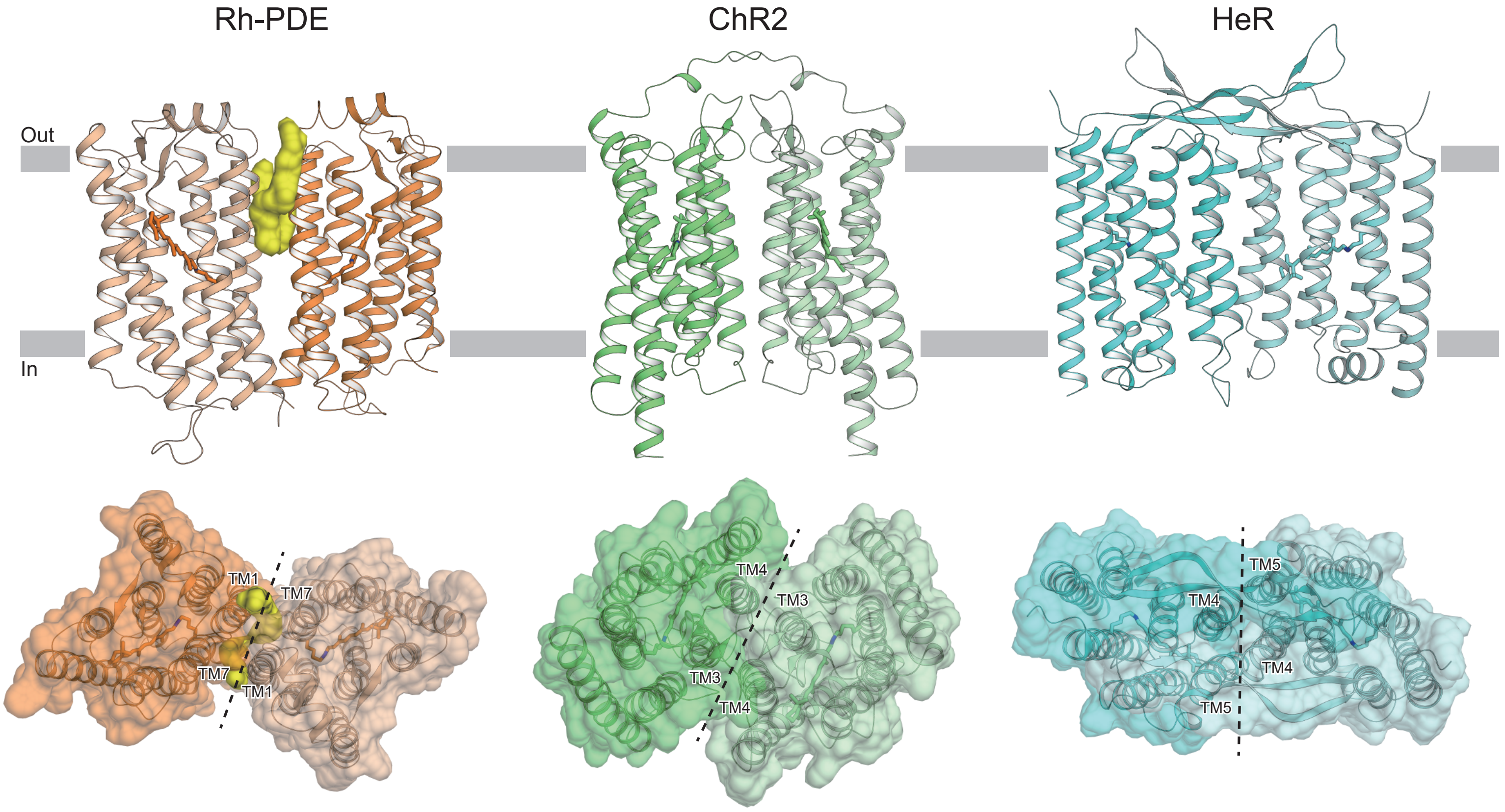

Supplementary Fig. 2 | Overall structural comparison with ChR2 and HeR. Overall structures of Rh-PDE (left), ChR2 (middle) and HeR (right), viewed from the membrane plane (upper) and extracellular side (lower). Monoolein molecules are colored yellow.

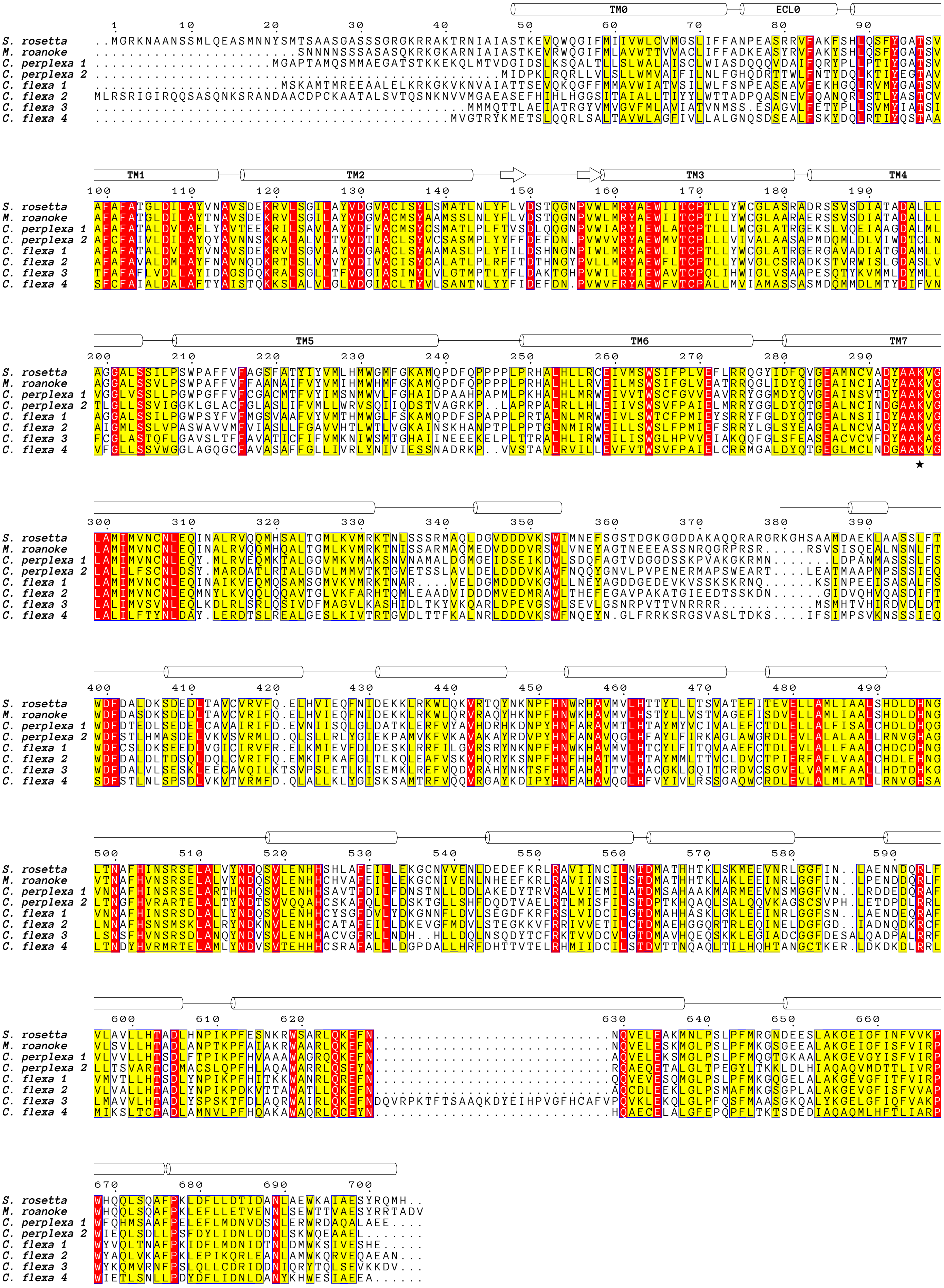

Supplementary Fig. 3 | Sequence alignment with Rh-PDE homologs. Amino acid sequence alignment of *Sr*Rh-PDE and other Rh-PDE homologs, from *Microstomoeca roanoke*, *Choanoeca perplexa*, and *Choanoeca flexa*. The star indicates the conserved lysine residues in TM7.

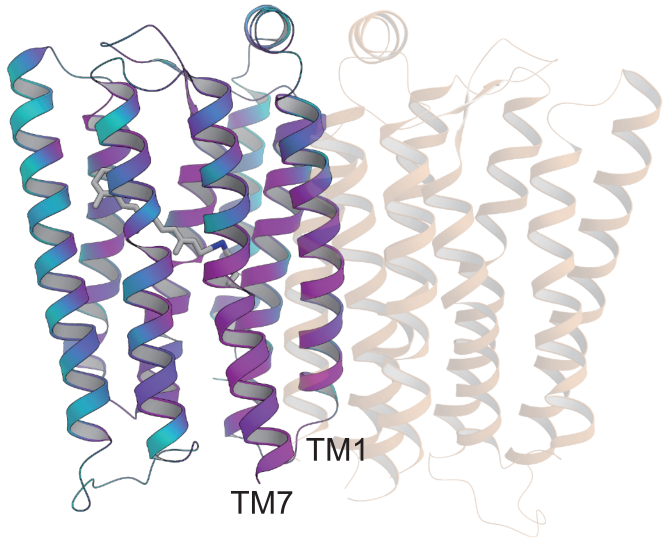

Supplementary Fig. 4 | Conservation of surface residues. Conservation of the surface residues of the Rh-PDE structure. The sequence conservation among seven Rh-PDE homologs was calculated using the ConSurf server (http://consurf.tau.ac.il) and is coloured from cyan (low) to maroon (high).

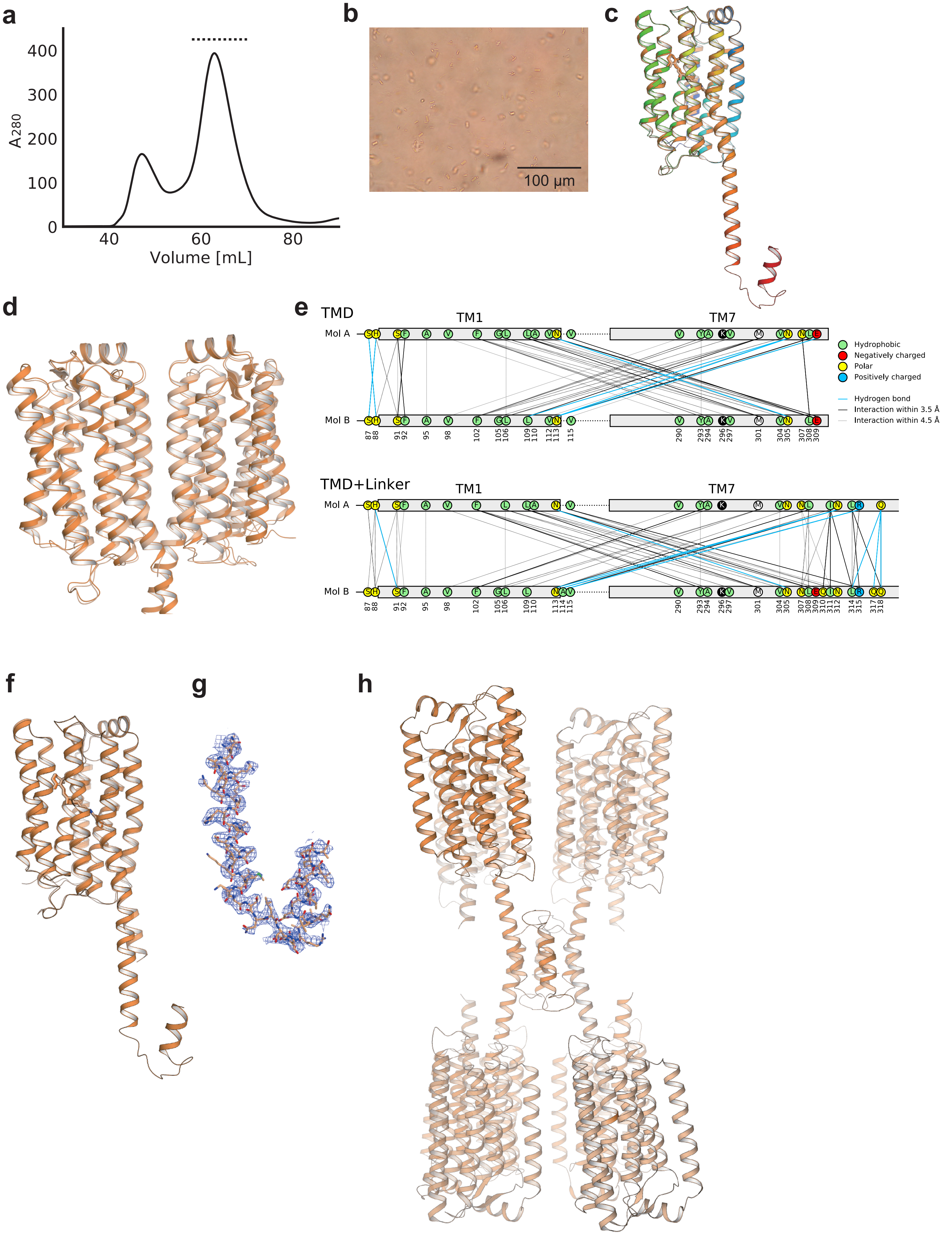
a
400
300
200
100
0
A280
40
60
80
Volume [mL]
b
100 µm
c
d
e
TMD
TM1
TM7
Mol A
Mol B
87 88 91 92 95 98 102 105 106 109 110 112 113 115 290 293 294 296 297 301 304 305 307 308 309
Hydrophobic
Negatively charged
Polar
Positively charged
Hydrogen bond
Interaction within 3.5 Å
Interaction within 4.5 Å
TMD+Linker
TM1
TM7
Mol A
Mol B
87 88 91 92 95 98 102 105 106 109 110 113 114 115 290 293 294 296 297 301 304 305 307 308 309 310 311 312 314 315 317 318
f
g
h

Supplementary Fig. 5 | Purification and crystallization of Rh-PDE TMD-Linker. a, Gel filtration chromatogram of the purified Rh-PDE TMD-Linker. Fractions with dashed lines were collected for the following crystallization. **b,** Crystals of Rh-PDE TMD-Linker. **c,** Structural comparison between Rh-PDE TMD and TMD-Linker. TMD and TMD-Linker are colored orange and rainbow, respectively. **d,** Dimer interface comparison between the TMD and TMD-Linker. TMD and TMD-Linker are colored light and dark orange, respectively. **e,** Distance mapping of Rh-PDE TMD and TMD-Linker. Interactions within 4.5 Å are shown in lines. **f,** Structural comparison between Rh-PDE TMD mol A and TMD-Linker mol B. TMD and TMD-Linker are colored light and dark orange, respectively. **g,** 2Fo-Fc map of the linker region of Rh-PDE TMD-Linker, contoured at 1.0σ. **h,** Crystal packing of Rh-PDE TMD-Linker.

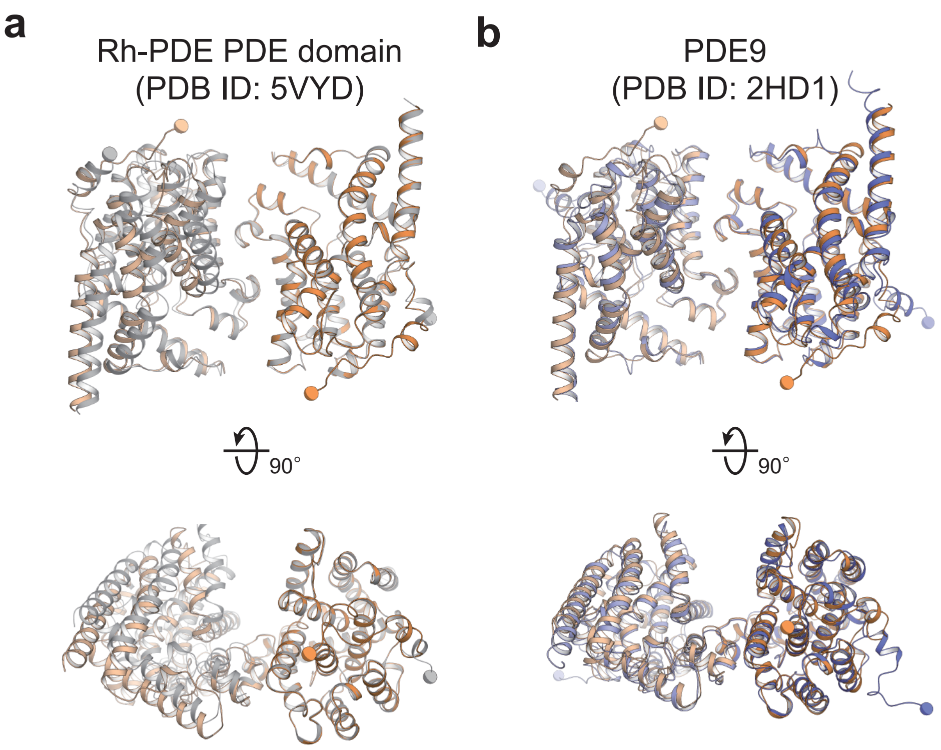

Supplementary Fig. 6 | Structural comparison of PDE domains. a, Structural comparison with the previous reported Rh-PDE PDE domain (PDB ID: 5VYD). Our structure and the previous structure are colored orange and gray, respectively. N-terminal residues of each structure are shown as spheres. **b,** Structural comparison with the PDE9 catalytic domain (PDB ID: 2HD1). Our structure and the PDE9 structure are colored orange and blue, respectively. N-terminal residues of each structure are shown as spheres.

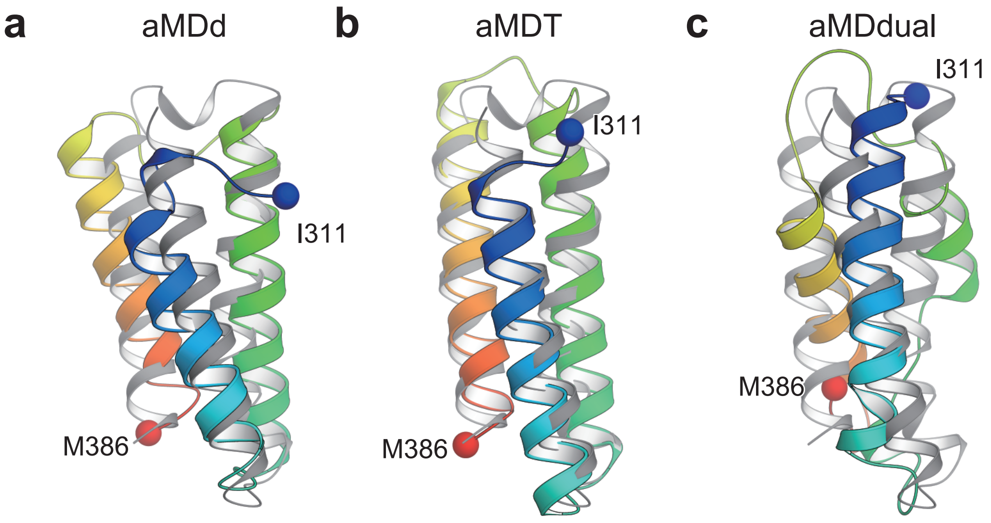

Supplementary Fig. 7 | Structural comparison of the linker of aMD simulations. a-c, Structur[al comparison between the linker model shown in Fig. 4e and the final structures of aMDd (**a**), aMDT (**b**) and aMDdual (**c**). The linker model and each aMD structure are colored gray and rainbow, respectively. Cα atoms of I311 and M386 are shown as spheres.

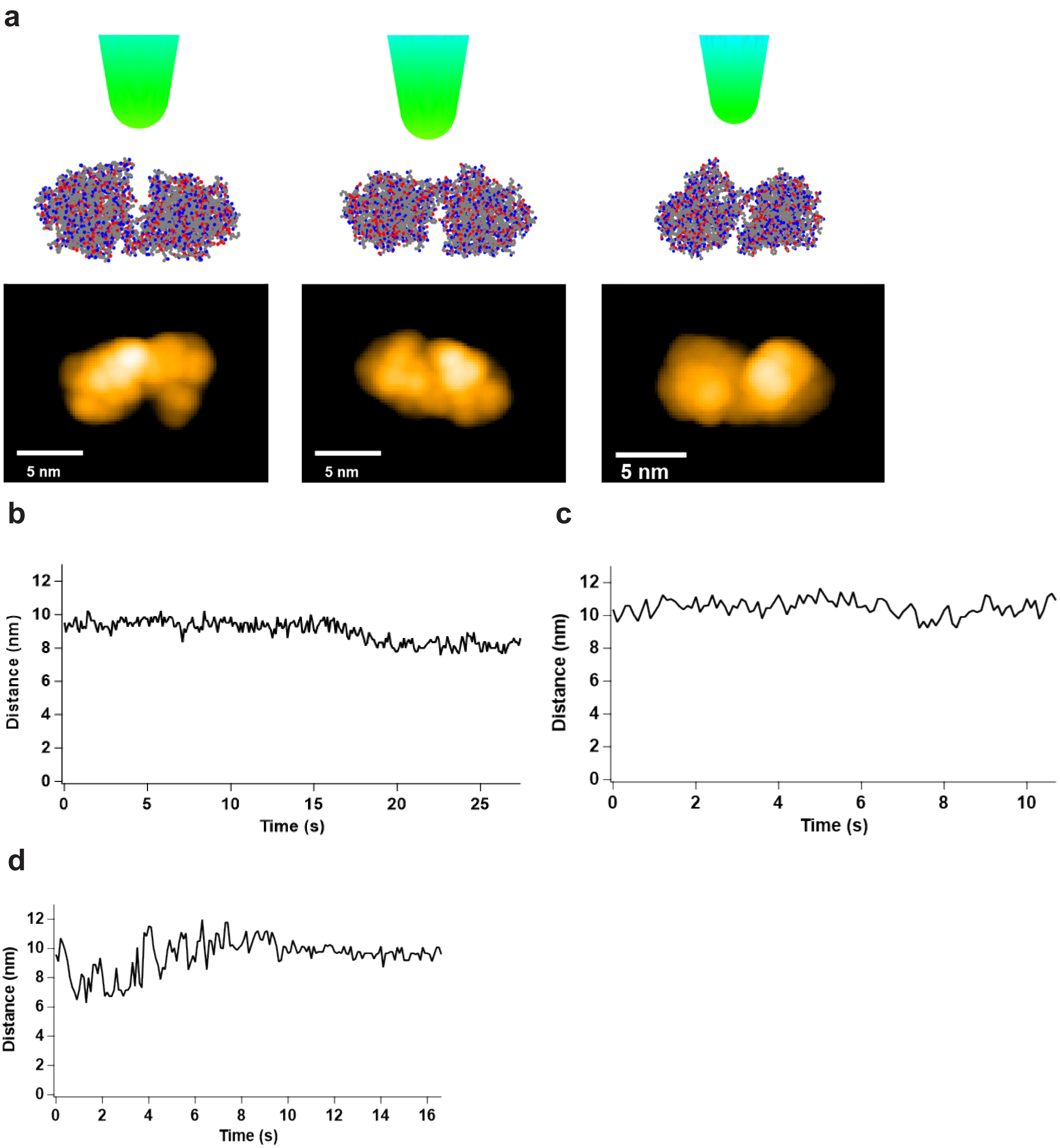

Supplementary Fig. 8 | HS-AFM. a, Simulation images of PDE dimers, from three viewpoints. **b-d,** Distance measurements between the centers of TMD and PDE domain in Supplementary Video 1-3.

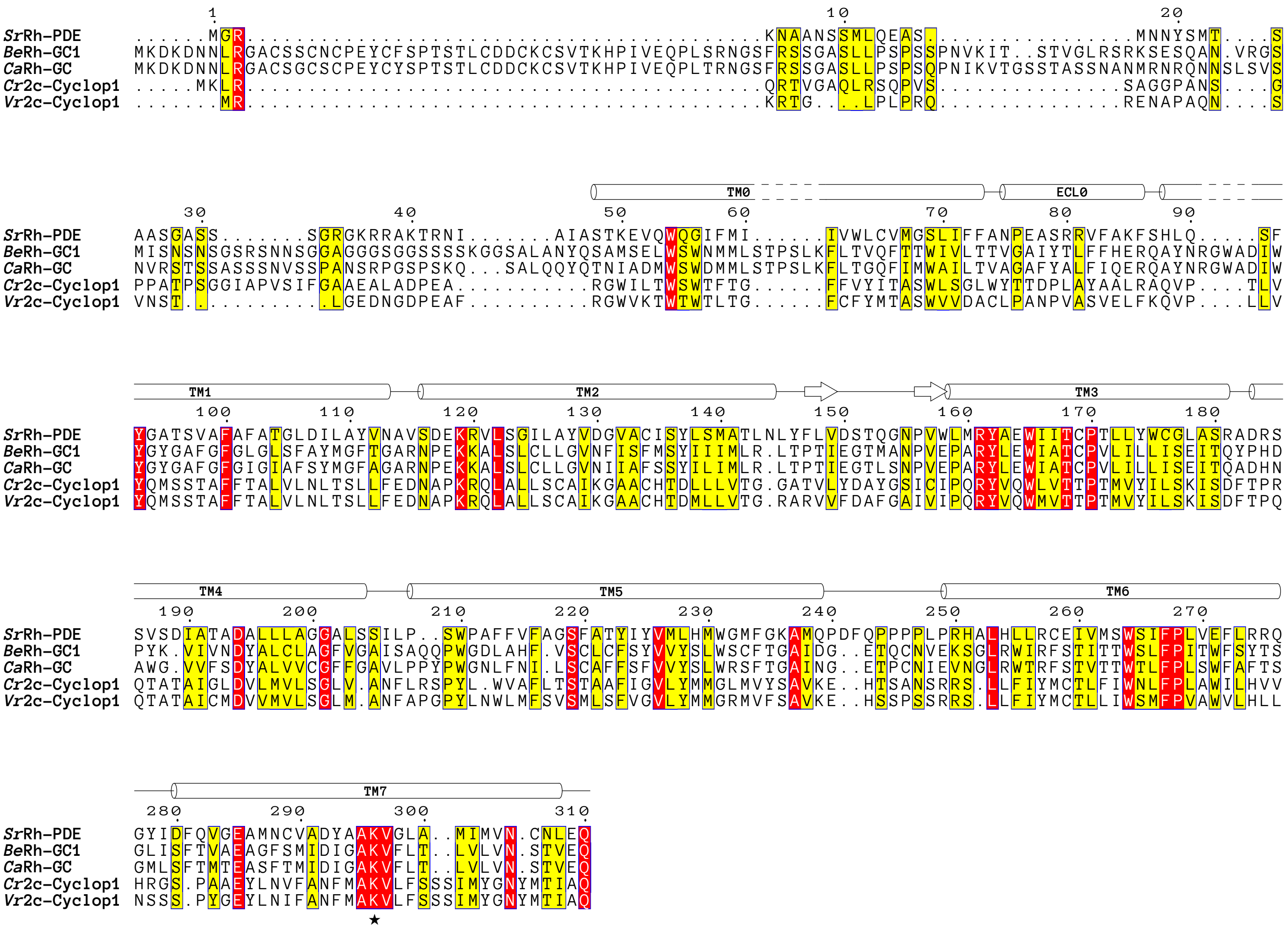

Supplementary Fig. 9 | Sequence alignment with rhodopsin enzymes. Amino acid sequence alignment of *Sr*Rh-PDE TMD and corresponding regions of rhodopsin enzymes, from *Blastocladiella emersonii* rhodopsin-guanylyl cyclase 1 (*Be*Rh-GC1), *Catenaria anguillulae* rhodopsin-guanylyl cyclase (*Ca*Rh-GC), *Chlamydomonas reinhardtii* two-component cyclase opsin 1 (*Cr*2c-Cyclop1) and *Volvox carteri* two-component cyclase opsin 1 (*Vr*2c-Cyclop1). The star indicates the conserved lysine residues in TM7.

Supplementary Table 1 | Data collection and refinement statistics.

	TMD	TMD-Linker	Linker-PDE
PDB ID	7CJ3	7D7Q	7D7P
**Data collection**			
Space group	$P2_12_12_1$	$I222$	$C2$
Cell dimensions			
a, *b*, *c* (Å)	65.55, 74.14, 117.38	76.30, 136.53, 206.54	117.2, 67.54, 56.89
α, β, γ (°)	90, 90, 90	90, 90, 90	90, 110.817, 90
Resolution (Å)*	49.11 - 2.6	49.40 - 3.5	47.53 - 2.1
	(2.76 - 2.6)	(3.71 - 3.5)	(2.18 - 2.1)
R_{meas}*	0.845 (13.2)	0.673 (2.868)	0.162 (0.798)
$<I/\sigma(I)>$ *	7.13 (0.76)	3.90 (1.04)	11.4 (2.5)
$CC_{1/2}$*	0.996 (0.609)	0.934 (0.393)	0.992 (0.660)
Completeness (%)*	99.9 (100)	98.1 (98.5)	90.7 (81.7)
Redundancy*	62.4 (57.0)	6.55 (6.18)	11.6 (8.7)
**Refinement**			
Resolution (Å)	49.11 - 2.6	49.40 - 3.5	47.53 - 2.1
No. reflections	18,193	13,712	22,066
R_{work} / R_{free}	0.2461 / 0.2946	0.2683 / 0.3145	0.1883 / 0.2397
No. atoms	4,402	4,611	2,814
Protein	4,052	4,485	2,600
Ligand/ion	334	126	38
Water	16	0	176
Averaged *B*-factors (Å^2)	40.43	33.37	27.78
Protein	39.35	33.48	26.99
Ligand/ion	54.37	29.29	42.23
Water	23.06		36.21
R.m.s. deviations from ideal			
Bond lengths (Å)	0.016	0.003	0.004
Bond angles (°)	1.81	0.61	0.88
Ramachandran plot			
Favored (%)	97.32	95.55	99.08
Allowed (%)	2.49	4.11	0.92
Outlier (%)	0.19	0.34	0

*Values in parentheses are for highest-resolution shell.
