# Supplementary material for: Structural insights into the mechanism of rhodopsin phosphodiesterase
Source: Nat Commun. 2020 Nov 5;11:5605. doi: 10.1038/s41467-020-19376-7 (PMC7644710; doi:10.1038/s41467-020-19376-7)
Supplement: Supplementary file 6 — Reporting Summary [file 41467_2020_19376_MOESM6_ESM.pdf]

nature research

Corresponding author(s): Osamu Nureki

Last updated by author(s): Oct 5, 2020

Reporting Summary

Nature Research wishes to improve the reproducibility of the work that we publish. This form provides structure for consistency and transparency in reporting. For further information on Nature Research policies, see our Editorial Policies and the Editorial Policy Checklist.

Statistics

For all statistical analyses, confirm that the following items are present in the figure legend, table legend, main text, or Methods section.

n/a	Confirmed	
☐	☒	The exact sample size (n) for each experimental group/condition, given as a discrete number and unit of measurement
☐	☒	A statement on whether measurements were taken from distinct samples or whether the same sample was measured repeatedly
☒	☐	The statistical test(s) used AND whether they are one- or two-sided<br>*Only common tests should be described solely by name; describe more complex techniques in the Methods section.*
☒	☐	A description of all covariates tested
☒	☐	A description of any assumptions or corrections, such as tests of normality and adjustment for multiple comparisons
☐	☒	A full description of the statistical parameters including central tendency (e.g. means) or other basic estimates (e.g. regression coefficient) AND variation (e.g. standard deviation) or associated estimates of uncertainty (e.g. confidence intervals)
☒	☐	For null hypothesis testing, the test statistic (e.g. F, t, r) with confidence intervals, effect sizes, degrees of freedom and P value noted<br>*Give P values as exact values whenever suitable.*
☒	☐	For Bayesian analysis, information on the choice of priors and Markov chain Monte Carlo settings
☒	☐	For hierarchical and complex designs, identification of the appropriate level for tests and full reporting of outcomes
☒	☐	Estimates of effect sizes (e.g. Cohen's d, Pearson's r), indicating how they were calculated

Our web collection on statistics for biologists contains articles on many of the points above.

Software and code

Policy information about availability of computer code

Data collection	ZOO as of June 2018, SHIKA as of June 2018
Data analysis	KAMO as of 2020, XDS Version Mar 15, 2019, Phenix 1.17, REFMAC 5.8, COOT 0.8.9, CueMol 2.2.3, PyMOL 2.3.0, PSIPRED, Rosetta 3.11, VMD 1.9.3, NAMD 2.13, Phaser 2.8.3, ASTRA software 7.1

For manuscripts utilizing custom algorithms or software that are central to the research but not yet described in published literature, software must be made available to editors and reviewers. We strongly encourage code deposition in a community repository (e.g. GitHub). See the Nature Research guidelines for submitting code & software for further information.

Data

Policy information about availability of data

All manuscripts must include a data availability statement. This statement should provide the following information, where applicable:

- Accession codes, unique identifiers, or web links for publicly available datasets
- A list of figures that have associated raw data
- A description of any restrictions on data availability

The atomic coordinates and structure factors of Rh-PDE have been deposited in the Protein Data Bank (PDB) (https://www.rcsb.org/) with accession codes 7CJ3 [http://doi.org/10.2210/pdb7CJ3/pdb] (TMD), 7D7Q [http://doi.org/10.2210/pdb7D7Q/pdb] (TMD-Linker) and 7D7P [http://doi.org/10.2210/pdb7D7P/pdb] (Linker-PDE). Source data of western blotting (Fig. 3c) are provided with this paper. Other data used in this study were available at NCBI with gene ID 16078606 [https://www.ncbi.nlm.nih.gov/gene/?term=16078606] (Rh-PDE gene) and PDB with accession codes 1C3W [http://doi.org/10.2210/pdb1C3W/pdb], 1M0M [http://doi.org/10.2210/pdb1M0M/pdb] (bacteriorhodopsin), 6EID [http://doi.org/10.2210/pdb6EID/pdb] (channelrhodopsin2), 2HD1 [http://doi.org/10.2210/pdb2HD1/pdb] (PDE9 catalytic domain), and 5VYD [http://doi.org/10.2210/pdb5VYD/pdb] (Rh-PDE PDE domain). All other data are available from the corresponding authors upon reasonable request.

Field-specific reporting

Please select the one below that is the best fit for your research. If you are not sure, read the appropriate sections before making your selection.

☒ Life sciences ☐ Behavioural & social sciences ☐ Ecological, evolutionary & environmental sciences

For a reference copy of the document with all sections, see nature.com/documents/nr-reporting-summary-flat.pdf

Life sciences study design

All studies must disclose on these points even when the disclosure is negative.

Sample size	Sample sizes were determined based on prior literature and best practices in the field; no statistical methods were used to predetermine sample size. The sample size was determined according to the previous study (ref 13).
Data exclusions	No data was excluded from the analysis.
Replication	All enzymatic activity measurements were performed repeatedly with the statements of the sample size in the text and methods section.
Randomization	There is no group allocation in the study, so no randomization is needed.
Blinding	There is no group allocation in the study, so no blinding is needed.

Reporting for specific materials, systems and methods

We require information from authors about some types of materials, experimental systems and methods used in many studies. Here, indicate whether each material, system or method listed is relevant to your study. If you are not sure if a list item applies to your research, read the appropriate section before selecting a response.

Materials & experimental systems

n/a	Involved in the study	
☐	☒	Antibodies
☐	☒	Eukaryotic cell lines
☒	☐	Palaeontology and archaeology
☒	☐	Animals and other organisms
☒	☐	Human research participants
☒	☐	Clinical data
☒	☐	Dual use research of concern

Methods

n/a	Involved in the study	
☒	☐	ChIP-seq
☒	☐	Flow cytometry
☒	☐	MRI-based neuroimaging

Antibodies

Antibodies used	RHO 1D4 Antibody - Antibody to Rhodopsin (University of British Columbia-UBC, Lot# 1019)
Validation	https://ubc.flintbox.com/#technologies/0f1ef64b-fa5d-4a58-9003-3e01f6f672a6

Eukaryotic cell lines

Policy information about cell lines

Cell line source(s)	HEK293 cells were from ThermoFisher Scientific (cat. no. R70507) for crystallization and HS-AFM observation, and from JCRB Cell Bank for the other experiments.
Authentication	None of the cell lines used have been authenticated.
Mycoplasma contamination	The cell lines were not tested for Mycoplasma contamination.
Commonly misidentified lines (See ICLAC register)	None of commonly misidentified lines were used in this study.
